# Electromagnetic force investigation of electromagnets with variable pole area in an electromagnetic diaphragm pump

**DOI:** 10.1371/journal.pone.0292685

**Published:** 2023-10-12

**Authors:** Yu Liao, Yinshui Liu, Jun Xing, Biao Chen, Lizhi Gao

**Affiliations:** 1 School of Mechanical Science and Engineering, Huazhong University of Science and Technology, Wuhan, China; 2 Shenzhen Academy of Inspection and Quarantine, Shenzhen, China; 3 Midea Group, Foshan, China; 4 Hubei Automobile Exhaust Emission Control and Basic Component Engineering Technology Research Center, Wuhan, China; COMSATS University Islamabad, PAKISTAN

## Abstract

Electromagnetic diaphragm pump is a kind of widely applied diaphragm pump that has promising sealing performance, simple structure and low power loss. Planar pole electromagnet is a significant component of the electromagnetic diaphragm pump. However, the sharply changing displacement-force characteristics of the planar pole electromagnet do not match the constant load characteristics of the electromagnetic diaphragm pump. Herein, an electromagnet with variable pole area is put forward. A theoretical relationship between structural parameters, the Ampere turns and the electromagnetic force of the electromagnet with variable pole area is determined by analyzing the equivalent magnetic circuit of the electromagnet with variable pole area. The experimental results imply that the initial electromagnetic force of the electromagnet with variable pole area is 32.51% larger than the planar pole electromagnet, the engaging electromagnetic force of the electromagnet with variable pole area is 22.3% smaller than the planar pole electromagnet and the displacement-force characteristics of the electromagnet with variable pole area match the constant load characteristics of the electromagnetic diaphragm pump.

## Introduction

Electromagnetic diaphragm pump is a type of diaphragm pump [1–5] that has quite a few advantages, such as simple structure and excellent sealing performance. Recent years have seen rapid development of the electromagnetic diaphragm pump. As an indispensable component of the electromagnetic diaphragm pump, planar pole electromagnet has been one of the most extensively applied electromagnets [6–9].

However, the sharply changing displacement-force characteristics of the planar pole electromagnet do not match the constant load characteristics of the electromagnetic diaphragm pump, that is, the initial electromagnetic force when the electromagnetic coil begins to be electrified is much smaller than the engaging electromagnetic force of the planar pole electromagnet. Moreover, the planar pole electromagnet will generate vibration, noise and impact when the initial electromagnetic force of the planar pole electromagnet is larger than the load required by the electromagnetic diaphragm pump.

Numerous researchers have suggested various methods to investigate and ameliorate the displacement-force characteristics of electromagnets [10–12]. S Wang and GY Xiong [13] constructed a non-working air gap between yoke and armature, and then connected it with the main working air gap in series. Yanping Hu [14] proposed an integral DC electromagnet with conical armature, investigated the effect of the conical armature angle on the electromagnetic force and conducted simulations of the DC electromagnet using Ansoft Maxwell software. However, this approach will increase the total magnetic reluctance and decrease the overall electromagnetic force of the proposed electromagnet. WZ Fu [15] established a mathematical model of an electromagnet used in a 630 kN multipoint forming machine, obtained the distribution curve for the electromagnetic force of this electromagnet and proposed an electromagnet with quasi-constant electromagnetic force. J Man and F Ding [16] compared the displacement-force characteristics of high-speed electromagnets with conical and planar pole. The initial electromagnetic force of the high-speed electromagnet with conical pole drops when the angle of the conical armature decreases. A Hashemi and PY Gharaei [17] put forward a magnetic equivalent circuit for an electromagnet with particular focus on fringing influence and leakage fluxes, and employed ANSYS Maxwell software to investigate the magnetic field, and employed MATLAB software to design and analyze the electromagnet, and conducted experiments to verify the precision of the proposed approach. The test results imply that the deviation between the simulation results and the experimental results is smaller than 2%. However, the aforementioned works have only paid attention to the engaging electromagnetic force, and have not improved the initial electromagnetic force of the planar pole electromagnet.

In this study, an electromagnet with variable pole area is put forward. A theoretical relationship between structural parameters, the Ampere turns and the electromagnetic force of the electromagnet with variable pole area is determined by analyzing the equivalent magnetic circuit of the electromagnet with variable pole area. Experiments are conducted to verify the results of theoretical calculations. The experimental results reveal that the initial electromagnetic force of the electromagnet with variable pole area is 32.51% larger than the planar pole electromagnet, the engaging electromagnetic force of the electromagnet with variable pole area is 22.3% smaller than that of the planar pole electromagnet and the displacement-force characteristics of the electromagnet with variable pole area match the constant load characteristics of the electromagnetic diaphragm pump.

The rest of this paper is organized as follows: the working philosophy and the structure of the electromagnet with variable pole area are presented in Section 2. Analysis on equivalent magnetic circuit of the electromagnet with variable pole area is given in Section 3. In Section 4, electromagnetic force calculations of the planar pole electromagnet are demonstrated. Experimental results will be demonstrated in Section 5. Finally, Section 6 concludes the work.

Structure and working philosophy of electromagnet with **variable pole area.**

**Fig 1:**
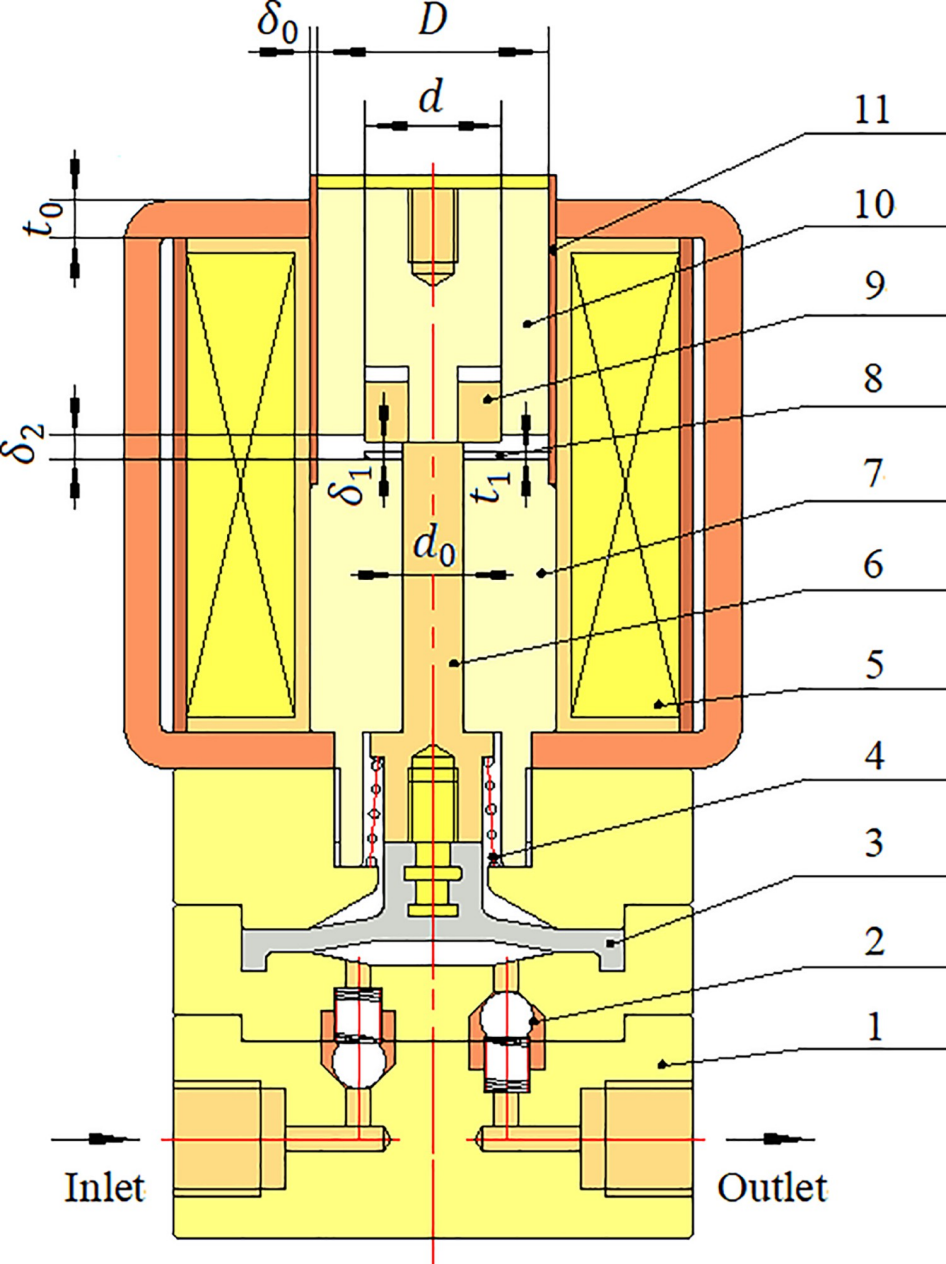
Cross-sectional scheme of electromagnetic diaphragm pump with electromagnet with variable pole area. 1- pump body; 2-single-direction valve; 3-diaphragm; 4-spring; 5-electromagnetic coil; 6-ejector pin; 7-the iron core; 8-magnetic isolation ring; 9-the inner armature; 10-the outer armature; 11-sleeve.

The electromagnetic diaphragm pump is composed of a pump body, ejector pin, spring, diaphragm, electromagnet with variable pole area and single-direction valves. The electromagnet with variable pole area mainly consists of the electromagnetic coil, iron core, magnetic isolation ring, the inner armature, the outer armature and sleeve as depicted in Fig 1 marked as 5, 7, 8, 9, 10, 11. The main parameters of the electromagnet with variable pole area are shown in Table 1.

**Table 1:** The main parameters of electromagnet with variable pole area.

Symbol	Parameter
*D*	Diameter of the outer armature.
*d*	Diameter of the inner armature.
*d*_0_	Diameter of ejector pin.
*t*_0_	Thickness of yoke.
*δ*_0_	Non-working air gap.
*δ*_2_	Initial working air gap between the outer armature and the iron core.
*δ*_1_	Initial working air gap between the inner armature and the iron core.
*δ*	Real-time working air gap between the outer armature and the iron core.
*δ*_n_	Real-time working air gap between the inner armature and the iron core.
*t*_1_	Thickness of magnetic isolation ring.
*F*_m_	Magnetic potential.
*I*_d_ *N*_d_	The Ampere turns.

In the electromagnet with variable pole area, the outer armature, the iron core and the inner armature are all made of electrical pure iron DT4, and the sleeve is made of stainless steel, and the magnetic isolation ring is made of copper and welded on the working end face of the iron core with laser. The function of the magnetic isolation ring is to maintain a certain magnetic reluctance between the inner armature and the iron core, and maintain a certain magnetic potential between the outer armature and the iron core in the process of armature engagement. The inner armature is installed in the central circular hole of the outer armature and can slide relative to the outer armature. The process of armature engagement can be divided into two stages: the initial stage and the end stage. Within the former, the electromagnetic force of the electromagnet with variable pole area is equal to the sum for the electromagnetic force of the inner armature and the electromagnetic force of the outer armature. When the inner armature contacts the magnetic isolation ring, the inner armature is restricted by the magnetic isolation ring, and the electromagnetic force of the electromagnet with variable pole area is equal to the electromagnetic force of the outer armature. There exist two working air gaps in the electromagnet with variable pole area: working air gap between the inner armature and the iron core, and working air gap between the outer armature and the iron core. The initial working air gap between the outer armature and the iron core when the electromagnetic coil begins to be energized is equal to the stroke of the electromagnetic diaphragm pump, and the working air gap between the inner armature and the iron core is smaller than the working air gap between the outer armature and the iron core in the initial stage.

## Analysis on equivalent magnetic circuit of electromagnet with variable pole area

**Fig 2:**
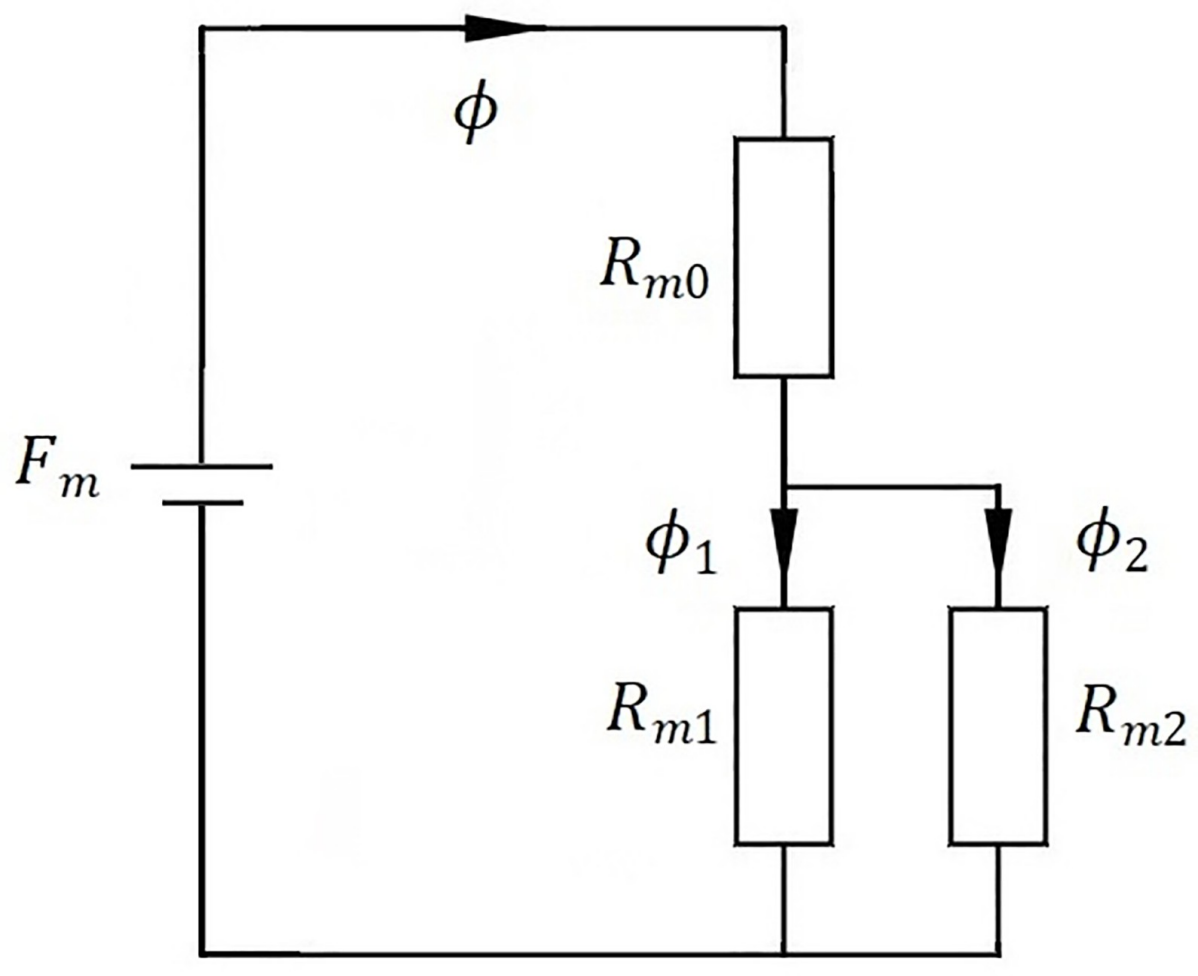
Equivalent magnetic circuit of electromagnet with variable pole area.

An equivalent magnetic circuit of the electromagnet with variable pole area is constructed to investigate the electromagnetic force of the electromagnet with variable pole area, as illustrated in Fig 2. In Fig 2, *F*_m_ is the magnetic potential of the electromagnet with variable pole area, *R*_m0_ is the magnetic reluctance of non-working air gap, *R*_m1_ is the magnetic reluctance of the working air gap between the inner armature and the iron core, *R*_m2_ is the magnetic reluctance of the working air gap between the outer armature and the iron core, *ϕ*, *ϕ*_1_ and *ϕ*_2_ are the total magnetic flux of the electromagnet with variable pole area, the magnetic flux in the working air gap between the inner armature and the iron core, and the magnetic flux in the working air gap between the outer armature and the iron core, respectively.

As manifested in Fig 2, the total magnetic reluctance of the electromagnet with variable pole area is composed of the magnetic reluctance for the total working air gap and the magnetic reluctance for the non-working air gap in series, and the magnetic reluctance of the total working air gap is composed of the magnetic reluctance for the working air gap between the inner armature and the iron core and the magnetic reluctance for the working air gap between the outer armature and the iron core in parallel.

### Calculations of flux area and magnetic reluctance

The flux area in non-working air gap is described as

$$S_{0}=\pi \left(\frac{t_{0}}{2}\times \frac{D}{2}\right)=\frac{\pi t_{0}D}{4}$$ (1)

where *t*_0_ is the thickness of the yoke and *D* is the diameter of the outer armature. The flux area in the working air gap between the inner armature and the iron core is expressed as

$$S_{1}=\frac{\pi (d^{2}-d_{0}^{2})}{4}$$ (2)

where *d* is the diameter of the inner armature and *d*_0_ is the diameter of the ejector pin in the electromagnet with variable pole area. The flux area in the working air gap between the outer armature and the iron core is calculated as

$$S_{2}=\frac{\pi (D^{2}-d^{2})}{4}$$ (3)

According to (1), the magnetic reluctance of the non-working air gap is computed as

$$R_{m0}=\frac{\delta_{0}}{\mu_{0}S_{0}}=\frac{4\delta_{0}}{\pi \mu_{0}t_{0}D}$$ (4)

where *δ*_0_ is non-working air gap of the electromagnet with variable pole area and *μ*_0_ is the vacuum permeability. As mentioned in Section 2, the difference between the real-time working air gap between the inner armature and the iron core and the real-time working air gap between the outer armature and the iron core is invariant in the initial stage, then the real-time working air gap between the inner armature and the iron core in the initial stage can be described as

$$\delta_{n}=\delta -(\delta_{2}-\delta_{1})$$ (5)

where *δ*_n_ is the real-time working air gap between the inner armature and the iron core, *δ* is the real-time working air gap between the outer armature and the iron core, *δ*_2_ is the initial working air gap between the outer armature and the iron core when the electromagnetic coil of the electromagnet with variable pole area begins to be energized, and *δ*_1_ is the initial working air gap between the inner armature and the iron core. According to (2) and (5), the magnetic reluctance of the working air gap between the inner armature and the iron core in the initial stage is presented by

$$R_{m1}=\frac{\delta_{n}}{\mu_{0}S_{1}}=\frac{4(\delta +\delta_{1}-\delta_{2})}{\pi \mu_{0}(d^{2}-d_{0}^{2})}$$ (6)

In the end stage, the inner armature has contacted the magnetic isolation ring, the working air gap between the inner armature and the iron core is invariant and equal to the thickness of the magnetic isolation ring, then the magnetic reluctance of the working air gap between the inner armature and the iron core in the end stage can be given as

$$R_{t1}=\frac{t_{1}}{\mu_{0}S_{1}}=\frac{4t_{1}}{\pi \mu_{0}(d^{2}-d_{0}^{2})}$$ (7)

where *t*_1_ is the thickness of the magnetic isolation ring. Furthermore, the magnetic reluctance of the working air gap between the outer armature and the iron core in the entire process of armature engagement can be written as follows:

$$R_{m2}=\frac{\delta }{\mu_{0}S_{2}}=\frac{4\delta }{\pi \mu_{0}(D^{2}-d^{2})}$$ (8)

By taking advantage of (6) and (8), the magnetic reluctance of the total working air gap in the initial stage is obtained.


$$R_{m}=\frac{R_{m1}R_{m2}}{R_{m1}+R_{m2}}=\frac{4\delta (\delta +\delta_{1}-\delta_{2})}{\pi \mu_{0}[(d^{2}-d_{0}^{2})\delta +(D^{2}-d^{2})(\delta +\delta_{1}-\delta_{2})]}$$ (9)

By taking advantage of (7) and (8), the magnetic reluctance of the total working air gap in the end stage is computed as follows:

$$R_{t}=\frac{R_{t1}R_{m2}}{R_{t1}+R_{m2}}=\frac{4t_{1}\delta }{\pi \mu_{0}[(d^{2}-d_{0}^{2})\delta +(D^{2}-d^{2})t_{1}]}$$ (10)

### Calculations of magnetic flux

The outer armature, the iron core and the inner armature in the electromagnet with variable pole area are all made of the electrical pure iron DT4. The magnetic reluctance of electrical pure iron DT4 is far less than the magnetic reluctance of air. If the magnetic reluctance of the electrical pure iron DT4 is ignored, the magnetic potential equation of the electromagnet with variable pole area in the initial stage can be expressed as

$$F_{m}=I_{d}N_{d}={\phi_{i}R}_{m0}+\phi_{i}R_{m}=\frac{4\delta_{0}\Phi_{i}}{\pi \mu_{0}t_{0}D}+\frac{4\delta (\delta +\delta_{1}-\delta_{2})\Phi_{i}}{\pi \mu_{0}[(d^{2}-d_{0}^{2})\delta +(D^{2}-d^{2})(\delta +\delta_{1}-\delta_{2})]}$$ (11)

where *F*_m_ is the magnetic potential and *I*_d_*N*_d_ is the Ampere turns of the electromagnet with variable pole area. According to the magnetic potential equation above, the total magnetic flux of the electromagnet with variable pole area in the initial stage is calculated as

$$\phi_{i}=\frac{F_{m}}{R_{m0}+R_{m}}=\frac{\pi \mu_{0}t_{0}D[(d^{2}-d_{0}^{2})\delta +(D^{2}-d^{2})(\delta +\delta_{1}-\delta_{2})]I_{d}N_{d}}{4[(d^{2}-d_{0}^{2})\delta +(D^{2}-d^{2})(\delta +\delta_{1}-\delta_{2})]\delta_{0}+4t_{0}D\delta (\delta +\delta_{1}-\delta_{2})}$$ (12)

According to the equivalent magnetic circuit manifested in Fig 2, in the initial stage, the magnetic flux in the working air gap between the inner armature and the iron core is described by

$$\phi_{i1}=\frac{R_{m2}\phi_{i}}{R_{m1}+R_{m2}}=\frac{\pi \mu_{0}t_{0}D\delta (d^{2}-d_{0}^{2})I_{d}N_{d}}{4\delta_{0}[(d^{2}-d_{0}^{2})\delta +(D^{2}-d^{2})(\delta +\delta_{1}-\delta_{2})]+4\delta (\delta +\delta_{1}-\delta_{2})t_{0}D}$$ (13)

In the initial stage, the magnetic flux in the working air gap between the outer armature and the iron core is presented by

$$\phi_{i2}=\frac{R_{m1}\phi_{i}}{R_{m1}+R_{m2}}=\frac{\pi \mu_{0}t_{0}D(D^{2}-d^{2})(\delta +\delta_{1}-\delta_{2})I_{d}N_{d}}{4[(d^{2}-d_{0}^{2})\delta +(D^{2}-d^{2})(\delta +\delta_{1}-\delta_{2})]\delta_{0}+4t_{0}D\delta (\delta +\delta_{1}-\delta_{2})}$$ (14)

According to the Kirchhoff’s second law of magnetic circuit, the magnetic potential equation of the electromagnet with variable pole area in the end stage is given as

$$F_{m}=I_{d}N_{d}=\phi_{e}R_{m0}+\phi_{e}R_{t}=\frac{4\delta_{0}\phi_{e}}{\pi \mu_{0}t_{0}D}+\frac{4t_{1}\delta \phi_{e}}{\pi \mu_{0}[(d^{2}-d_{0}^{2})\delta +(D^{2}-d^{2})t_{1}]}$$ (15)

Then the total magnetic flux of the electromagnet with variable pole area in the end stage can be determined accordingly.


$$\phi_{e}=\frac{F_{m}}{R_{m0}+R_{t}}=\frac{\pi \mu_{0}t_{0}D\mu_{0}[(d^{2}-d_{0}^{2})\delta +(D^{2}-d^{2})t_{1}]I_{d}N_{d}}{4\delta_{0}[(d^{2}-d_{0}^{2})\delta +(D^{2}-d^{2})t_{1}]+4t_{1}\delta t_{0}D}$$ (16)

In the end stage, the magnetic flux in the working air gap between the inner armature and the iron core is described as

$$\phi_{e1}=\frac{R_{m2}\phi_{e}}{R_{t1}+R_{m2}}=\frac{\pi \mu_{0}t_{0}D\delta (d^{2}-d_{0}^{2})I_{d}N_{d}}{4\delta_{0}[(d^{2}-d_{0}^{2})\delta +(D^{2}-d^{2})t_{1}]+4t_{1}\delta t_{0}D}$$ (17)

By taking advantage of (16), the magnetic flux in the working air gap between the outer armature and the iron core when the inner armature touches the magnetic isolation ring is computed as

$$\phi_{e2}=\frac{R_{t1}\phi_{e}}{R_{t1}+R_{m2}}=\frac{\pi \mu_{0}t_{0}t_{1}D(D^{2}-d^{2})I_{d}N_{d}}{4[(d^{2}-d_{0}^{2})\delta +(D^{2}-d^{2})t_{1}]\delta_{0}+4t_{0}t_{1}D\delta }$$ (18)

When the outer armature is overwhelmingly close to the iron core, the working air gap between the outer armature and the iron core is exceedingly tiny, the magnetic flux density of the outer armature will be saturated. Because the outer armature, the iron core and the inner armature in the electromagnet with variable pole area are all made of electrical pure iron DT4, the saturated magnetic flux of the electromagnet with variable pole area is determined according to the saturated magnetic flux density of electrical pure iron DT4.


$$\phi_{ms}=B_{s}S_{2}=\frac{\pi (D^{2}-d^{2})B_{s}}{4}$$ (19)

where *B*_s_ is the saturated magnetic flux density of electrical pure iron DT4. The working air gap between the outer armature and the iron core when the magnetic flux density of the outer armature begins to be saturated can be obtained by combining (18) and (19).


$$\delta_{ms}=\frac{\pi \mu_{0}t_{0}t_{1}D(D^{2}-d^{2})I_{d}N_{d}-\pi (D^{2}-d^{2})B_{s}(D^{2}-d^{2})t_{1}\delta_{0}}{\pi (D^{2}-d^{2})B_{s}[t_{0}t_{1}D+(d^{2}-d_{0}^{2})\delta_{0}]}$$ (20)

### Calculations of electromagnetic force

The Maxwell formula of electromagnetic force can be employed to calculate the electromagnetic force of the electromagnet with variable pole area. By substituting (13) into the Maxwell formula of electromagnetic force, the electromagnetic force of the inner armature in the initial stage is written as

$$F_{i1}=\frac{\phi_{i1}^{2}}{2\mu_{0}S_{1}}=\frac{\mu_{0}{\pi (t_{0}D\delta I_{d}N_{d})}^{2}(d^{2}-d_{0}^{2})}{8{\left\{\delta_{0}[(d^{2}-d_{0}^{2})\delta +(D^{2}-d^{2})(\delta +\delta_{1}-\delta_{2})]+\delta (\delta +\delta_{1}-\delta_{2})t_{0}D\right\}}^{2}}$$ (21)

By taking advantage of (14), the electromagnetic force of the outer armature in the initial stage is given as

$$F_{i2}=\frac{\phi_{i2}^{2}}{2\mu_{0}S_{2}}=\frac{\mu_{0}\pi {\left[t_{0}D(\delta +\delta_{1}-\delta_{2})I_{d}N_{d}\right]}^{2}(D^{2}-d^{2})}{8{\left\{\left[(d^{2}-d_{0}^{2})\delta +(D^{2}-d^{2})(\delta +\delta_{1}-\delta_{2})]\delta_{0}+t_{0}D\delta (\delta +\delta_{1}-\delta_{2}\right)\right\}}^{2}}$$ (22)

In the initial stage, the electromagnetic force of the electromagnet with variable pole area is equal to the sum for the electromagnetic force of the outer armature and the electromagnetic force of the inner armature, which is expressed as

$$F_{i}=F_{i1}+F_{i2}=\frac{{\left[\left(\delta +\delta_{1}-\delta_{2}\right)\right]}^{2}(D^{2}-d^{2})}{8{\left\{\left[(d^{2}-d_{0}^{2})\delta +(D^{2}-d^{2})(\delta +\delta_{1}-\delta_{2})]\delta_{0}+t_{0}D\delta (\delta +\delta_{1}-\delta_{2}\right)\right\}}^{2}}$$ (23)

The electromagnetic force of the inner armature in the end stage is determined according to (17).


$$F_{e1}=\frac{\phi_{e1}^{2}}{2\mu_{0}S_{1}}=\frac{\pi \mu_{0}{\left(t_{0}D\delta I_{d}N_{d}\right)}^{2}(d^{2}-d_{0}^{2})}{8{\left\{\delta_{0}[(d^{2}-d_{0}^{2})\delta +(D^{2}-d^{2})t_{1}]+t_{1}\delta t_{0}D\right\}}^{2}}$$ (24)

According to the working philosophy of the electromagnet with variable pole area, the electromagnetic force of the electromagnet with variable pole area in the end stage is equal to the electromagnetic force of the outer armature, which is obtained by substituting (18) into the Maxwell formula of electromagnetic force.


$$F_{2}=F_{e2}=\frac{\phi_{e2}^{2}}{2\mu_{0}S_{2}}=\frac{\pi \mu_{0}{\left(t_{0}t_{1}DI_{d}N_{d}\right)}^{2}(D^{2}-d^{2})}{8{\left\{[(d^{2}-d_{0}^{2})\delta +(D^{2}-d^{2})t_{1}]\delta_{0}+t_{0}t_{1}D\delta \right\}}^{2}}$$ (25)

The electromagnetic force of the outer armature when the magnetic flux density of the outer armature is saturated is expressed as

$$F_{2s}=\frac{\phi_{ms}^{2}}{2\mu_{0}S_{2}}=\frac{\pi (D^{2}-d^{2})B_{s}^{2}}{8\mu_{0}}$$ (26)

## Calculations for electromagnetic force of planar pole electromagnet

The flux area of non-working air gap in the planar pole electromagnet is calculated as

$$S_{np}=\pi \left(\frac{t_{f}}{2}\times \frac{D_{1}}{2}\right)=\frac{\pi t_{f}D_{1}}{4}$$ (27)

where *t*_f_ is the thickness of yoke in the planar pole electromagnet and *D*_1_ is the diameter of the armature in the planar pole electromagnet. According to (27), the magnetic reluctance of non-working air gap in the planar pole electromagnet is presented by

$$R_{np}=\frac{\delta_{f}}{\mu_{0}S_{np}}=\frac{\delta_{f}}{\pi \mu_{0}t_{f}D_{1}}$$ (28)

where *δ*_f_ is the non-working air gap of the planar pole electromagnet. The flux area of working air gap in the planar pole electromagnet is described by

$$S_{mp}=\frac{\pi (D_{1}^{2}-d_{1}^{2})}{4}$$ (29)

where *d*_1_ is the diameter of the ejector pin in the planar pole electromagnet. By taking advantage of (29), the magnetic reluctance of the working air gap in the planar pole electromagnet is given as

$$R_{mp}=\frac{\delta_{p}}{\mu_{0}S_{mp}}=\frac{4\delta_{p}}{\pi \mu_{0}(D_{1}^{2}-d_{1}^{2})}$$ (30)

where 0≤ *δ*_p_≤ *δ*_m_ is the real-time working air gap of the planar pole electromagnet and *δ*_m_ is the initial working air gap when the electromagnetic coil in the planar pole electromagnet begins to be energized. The magnetic potential equation of the planar pole electromagnet is expressed as

$$F_{mp}=I_{p}N_{p}=\phi_{p}R_{np}+\phi_{p}R_{mp}=\frac{4\delta_{f}\phi_{p}}{\pi \mu_{0}t_{f}D_{1}}+\frac{4\delta_{p}\phi_{p}}{\pi \mu_{0}(D_{1}^{2}-d_{1}^{2})}$$ (31)

where *F*_mp_ is the magnetic potential and *I*_p_*N*_p_ is the Ampere turns of the planar pole electromagnet. According to (31), the magnetic flux of the planar pole electromagnet is written as

$$\phi_{p}=\frac{F_{mp}}{R_{np}+R_{mp}}=\frac{\pi \mu_{0}{t_{f}D}_{1}(D_{1}^{2}-d_{1}^{2})I_{p}N_{p}}{{4\delta }_{f}(D_{1}^{2}-d_{1}^{2})+4\delta_{p}{t_{f}D}_{1}}$$ (32)

When the armature is extremely close to the iron core in the planar pole electromagnet, the working air gap of the planar pole electromagnet is quite small, the magnetic flux density of the armature will be saturated. Because the armature and the iron core in the planar pole electromagnet are made of electrical pure iron DT4, the saturated magnetic flux of the planar pole electromagnet is computed as

$$\phi_{ps}=B_{s}S_{mp}=\frac{\pi (D_{1}^{2}-d_{1}^{2})B_{s}}{4}$$ (33)

Then the working air gap of the planar pole electromagnet when magnetic flux density of the armature begins to be saturated can be obtained by combining (32) and (33).


$$\delta_{ps}=\frac{\mu_{0}{t_{f}D}_{1}(D_{1}^{2}-d_{1}^{2})I_{p}N_{p}-\delta_{f}(D_{1}^{2}-d_{1}^{2})(D_{1}^{2}-d_{1}^{2})B_{s}}{(D_{1}^{2}-d_{1}^{2})B_{s}{t_{f}D}_{1}}$$ (34)

By substituting (32) into the Maxwell formula of electromagnetic force, the electromagnetic force of the planar pole electromagnet is calculated as

$$F_{p}=\frac{\phi_{p}^{2}}{2\mu_{0}S_{mp}}=\frac{\pi \mu_{0}{\left({t_{f}D}_{1}I_{p}N_{p}\right)}^{2}(D_{1}^{2}-d_{1}^{2})}{8{\left[{4\delta }_{f}(D_{1}^{2}-d_{1}^{2})+\delta_{p}{t_{f}D}_{1}\right]}^{2}}$$ (35)

## Experimental results

An experimental equipment and an electromagnet prototype with variable pole area were designed and fabricated to measure the electromagnetic force of the electromagnet with variable pole area and verify the results of theoretical calculations. The parameters of the electromagnet prototype with variable pole area are manifested in Table 2. The thicknesses for the yoke in the electromagnet prototype with variable pole area and the planar pole electromagnet are the same, and the diameters of the ejector pin in the electromagnet prototype with variable pole area and the planar pole electromagnet are the same, i.e, *t*_0_ = *t*_*f*_, *d*_0_ = *d*_1_. Besides, the Ampere turns and non-working air gaps of the electromagnet prototype with variable pole area and the planar pole electromagnet are the same, i.e, *I*_*d*_*N*_*d*_ = I_p_*N*_*p*_, *δ*_0_ = *δ*_*f*_. The diameter of the outer armature in the electromagnet prototype with variable pole area is equal to the diameter of the armature in the planar pole electromagnet, and the initial working air gap between the outer armature and the iron core of the electromagnet prototype with variable pole area is equal to the initial working air gap of the planar pole electromagnet, i.e, *D* = *D*_1_, *δ*_2_ = *δ*_*m*_.

**Table 2:** The parameters of electromagnet prototype with variable pole area.

Parameters	Value
Diameter of the outer armature, *D*_0_	16 mm
Diameter of the inner armature, *d*	8 mm
Diameter of ejector pin, *d*_0_	4 mm
Thickness of yoke, *t*_0_	3 mm
Non-working air gap, *δ*_0_	1 mm
Initial working air gap between the outer armature and the iron core, *δ*_2_	2 mm
Initial working air gap between the inner armature and the iron core, *δ*_1_	1.4 mm
Thickness of magnetic isolation ring, *t*_1_	0.6 mm
The Ampere turns, *I*_d_*N*_d_	1450 A

**Fig 3:**
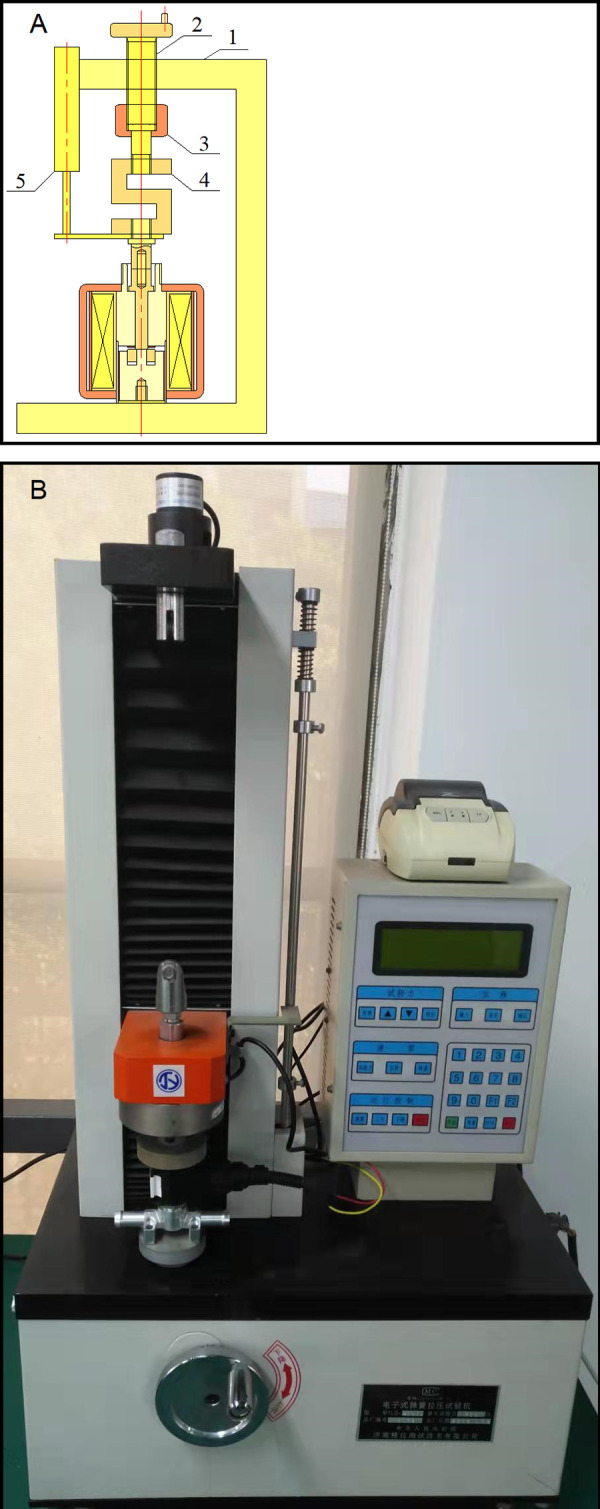
The experimental equipment (A) Schematic graph: 1-frame; 2-adjusting handwheel; 3-unfixed joint; 4-tension sensor; 5-displacement sensor (B) Photo.

The experiment was conducted using a spring tester, as depicted in Fig 3. The type of the spring tester is TLD-200. The spring tester mainly consists of a frame, tension sensor, unfixed joint, DC power supply, monitor, displacement sensor and adjusting handwheel. The parameters of the tension sensor and the displacement sensor are illustrated in Table 3. The displacement sensor is used to test the real-time working air gap between the outer armature and the iron core in the electromagnet prototype with variable pole area. The function of the tension sensor is to measure the electromagnetic force of the electromagnet prototype with variable pole area. The adjusting handwheel is employed to adjust the working air gap between the outer armature and the iron core. During the experiment, the outer armature of the electromagnet prototype with variable pole area is fixed to the frame, and the iron core is connected with the displacement sensor and tension sensor through the unfixed joint, and the working air gap between the outer armature and the iron core in the electromagnet prototype with variable pole area is adjusted according to the value tested by the displacement sensor. When testing the electromagnetic force of the electromagnet prototype with variable pole area with the tension sensor, the working air gap between the outer armature and the iron core is maintained at 0, 0.1, 0.2, 0.4, 0.6, 0.8, 1, 1.2, 1.4, 1.6, 1.8 and 2 mm, respectively.

**Table 3:** The parameters of the displacement sensor and the tension sensor.

Parameters	Value
Measuring range of the tension sensor	0–200 N
Measuring accuracy of the tension sensor	0.1 N
Measuring range of the displacement sensor	0–400 mm
Measuring accuracy of the displacement sensor	0.02 mm

**Fig 4:**
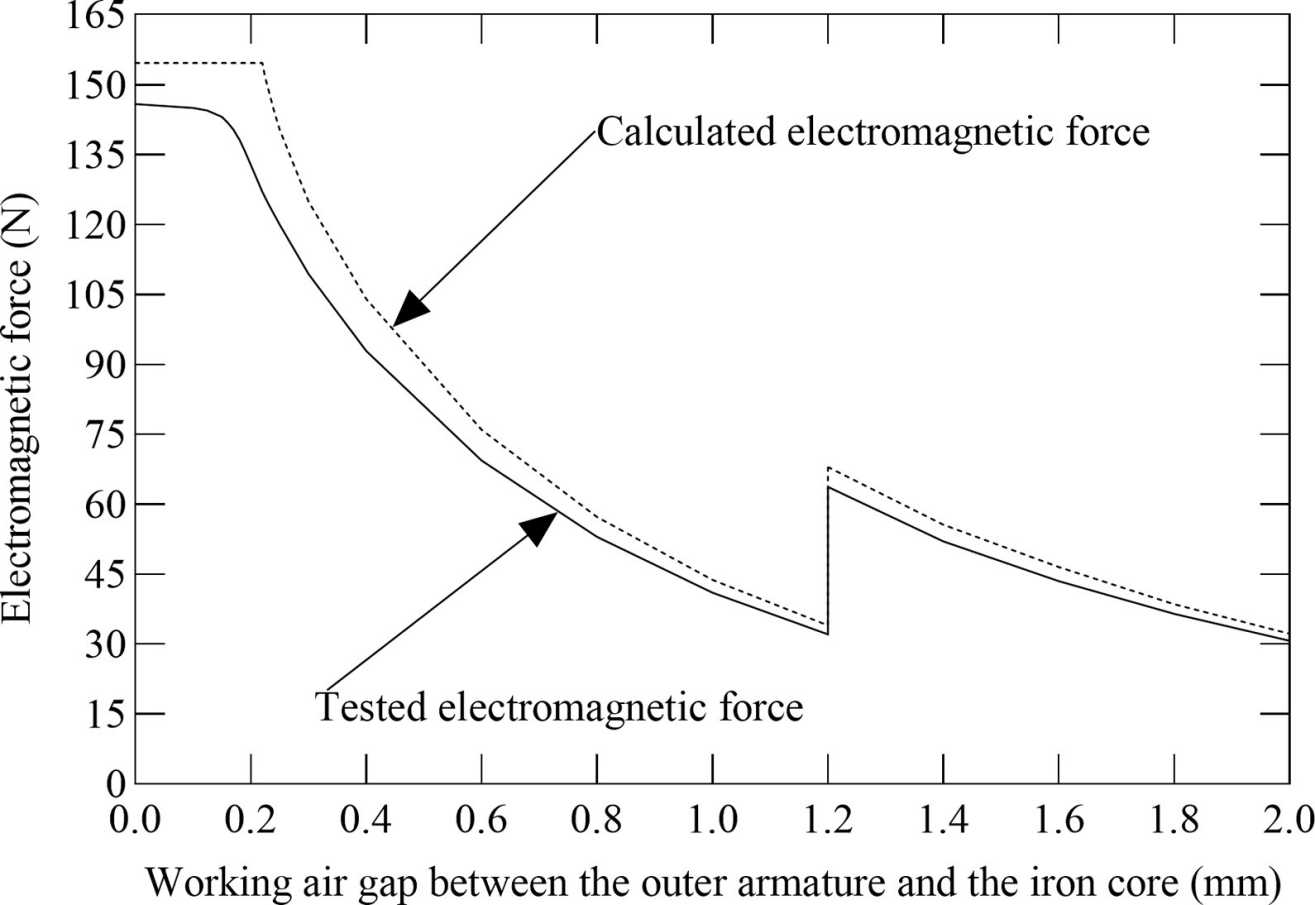
Comparison of the electromagnetic force between the calculations results with the experimental results at different working air gap between the outer armature and the iron core.

Fig 4 shows the electromagnetic force of the electromagnet with variable pole area varying with the working air gap between the outer armature and the iron core solution obtained by the theoretical calculations and by the experiment. In Fig 4, the solid line represents the tested electromagnetic force curve of the electromagnet with variable pole area, and the dotted line represents the calculated electromagnetic force curve of the electromagnet with variable pole area. It can be observed in Fig 4 that both the calculated electromagnetic force and the tested electromagnetic force of the electromagnet with variable pole area increase by decreasing the working air gap between the outer armature and the iron core. Besides, the tested electromagnetic force is smaller in comparison to the calculated electromagnetic force of the electromagnet with variable pole area. This is because leakage fluxes inside the electromagnetic coil are ignored in the theoretical calculation. The calculated electromagnetic force of the electromagnet with variable pole area no longer increases by decreasing the working air gap between the outer armature and the iron core when the working air gap between the outer armature and the iron core is 0.22 mm, which is because the magnetic flux density of the outer armature becomes saturated at this moment. The change rate for the tested electromagnetic force of the electromagnet with variable pole area begins to decrease when the working air gap between the outer armature and the iron core is 0.2 mm, and the tested electromagnetic force still slowly increases by reducing the working air gap between the outer armature and the iron core, and no longer increases when the working air gap between the outer armature and the iron core is 0.1 mm. This testifies that the actual magnetic flux density of the outer armature is smaller than the calculated magnetic flux density of the outer armature and there exist leakage fluxes inside the electromagnetic coil. It also can be found in Fig 4 that both the tested electromagnetic force and the calculated electromagnetic force of the electromagnet with variable pole area drop abruptly when the working air gap between the outer armature and the iron core is 1.2 mm, and then carry on increasing by declining the working air gap between the outer armature and the iron core, which is because the inner armature touches the magnetic isolation ring and can not carry on moving, the electromagnetic force of the inner armature is offset by the magnetic isolation ring, and the effective magnetic pole area of the electromagnet with variable pole area declines abruptly at this time. The maximum difference between the tested electromagnetic force and the calculated electromagnetic force of the electromagnet with variable pole area is approximately 27.65 N, and the results of theoretical calculations show agreement with the experimental results.

**Fig 5:**
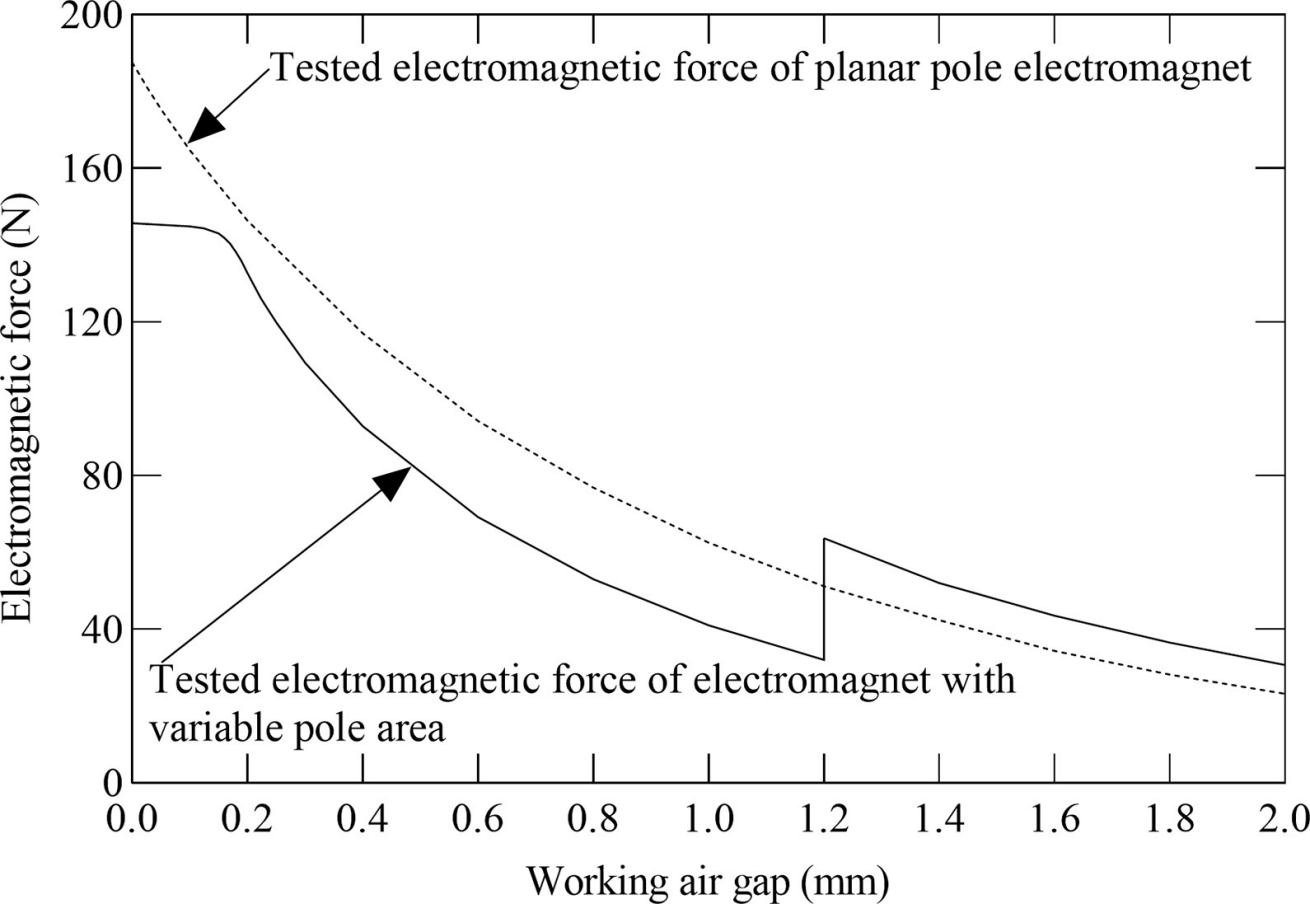
The effect of working air gap on the tested electromagnetic forces of electromagnet with variable pole area and planar pole electromagnet.

Fig 5 illustrates the tested electromagnetic forces of the electromagnet with variable pole area and the planar pole electromagnet changing with the working air gap. In Fig 5, the dotted line represents the tested electromagnetic force curve of the planar pole electromagnet versus the real-time working air gap, and the solid line represents the tested electromagnetic force curve of the electromagnet with variable pole area versus the real-time working air gap between the outer armature and the iron core. It can be observed in Fig 5 that the tested electromagnetic force of the electromagnet with variable pole area declines suddenly when the working air gap between the outer armature and the iron core is 1.2 mm, and continues to increase by decreasing the working air gap between the outer armature and the iron core afterward. This is because the inner armature contacts the magnetic isolation ring and can not continue to move, and the effective magnetic pole area of the electromagnet with variable pole area drops abruptly, and the electromagnetic force of the electromagnet with variable pole area is equal to the electromagnetic force of the outer armature, and the electromagnetic force of the inner armature is offset by the magnetic isolation ring at this time. Moreover, it also can be found in Fig 5 that the initial electromagnetic force of the electromagnet with variable pole area is 32.51% bigger than the initial electromagnetic force of the planar pole electromagnet, and the engaging electromagnetic force of the electromagnet with variable pole area is 22.3% smaller in comparison with the engaging electromagnetic force of the planar pole electromagnet. The ratio for the engaging electromagnetic force to the initial electromagnetic force of the electromagnet with variable pole area is 4.75, and the ratio for the engaging electromagnetic force to the initial electromagnetic force of the planar pole electromagnet is 8.1. The experimental results in Fig 5 suggest that the electromagnet with variable pole area has a bigger initial electromagnetic force and smaller engaging electromagnetic force than that of the planar pole electromagnet when the structural parameters and the Ampere turns are the same, and the displacement-force characteristics of the electromagnet with variable pole area match the constant load characteristics of the electromagnetic diaphragm pump.

**Fig 6:**
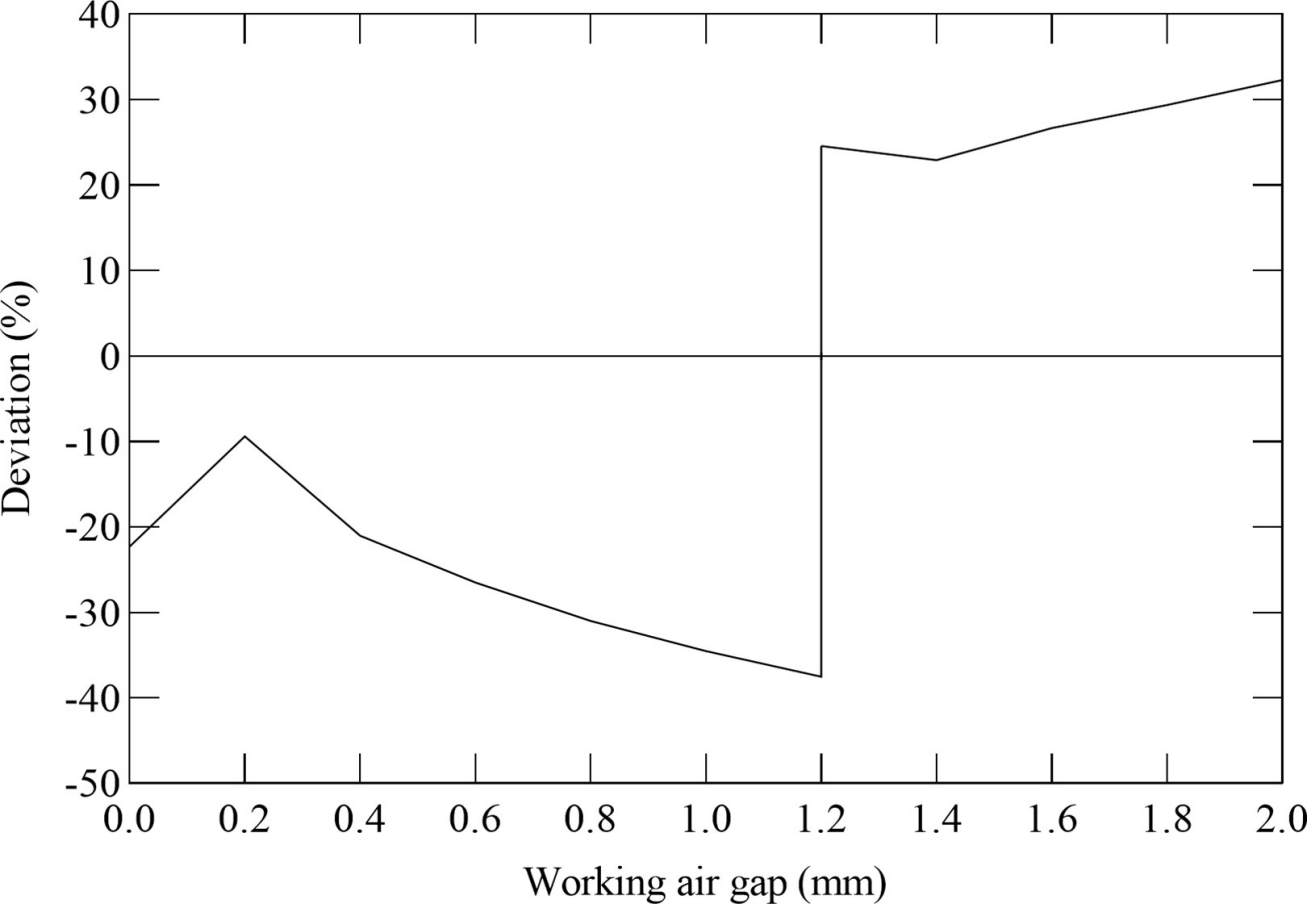
The influence of working air gap on the deviation between the tested electromagnetic force of electromagnet with variable pole area and the tested electromagnetic force of planar pole electromagnet.

Fig 6 illustrates the deviation between the tested electromagnetic force of the electromagnet with variable pole area and the tested electromagnetic force of the planar pole electromagnet changing with the working air gap.

## Conclusion

In this study, a kind of electromagnet with variable pole area is put forward. A theoretical relationship between structural parameters, the Ampere turns and the electromagnetic force of the electromagnet with variable pole area is ascertained by analyzing the equivalent magnetic circuit of the electromagnet with variable pole area. Experiments are carried out to verify the results of theoretical calculations. The experimental results imply that the engaging electromagnetic force of the electromagnet with variable pole area is 22.3% smaller than the planar pole electromagnet, and the initial electromagnetic force of the electromagnet with variable pole area is 32.51% larger than the planar pole electromagnet and the displacement-force characteristics of the electromagnet with variable pole area match the constant load characteristics of the electromagnetic diaphragm pump. The displacement-force characteristics of the electromagnet with variable pole area can be ameliorated, which is the object of our future work.

## Supporting information

S1 File Comparison of the electromagnetic force between the calculations results with the experimental results at different working air gap between the outer armature and the iron core. (XLSX)

S2 File The effect of working air gap on the tested electromagnetic forces of electromagnet with variable pole area and planar pole electromagnet. (XLSX)

S3 File The effect of working air gap on the deviation between the tested electromagnetic force of electromagnet with variable pole area and the tested electromagnetic force of planar pole electromagnet. (XLSX)
